# Online communication as a window to conspiracist worldviews

**DOI:** 10.3389/fpsyg.2015.00836

**Published:** 2015-06-17

**Authors:** Michael J. Wood, Karen M. Douglas

**Affiliations:** ^1^Department of Psychology, University of WinchesterWinchester, UK; ^2^School of Psychology, University of KentCanterbury, UK

**Keywords:** conspiracy theories, conspiracy mentality, computer-mediated communication, persuasion, projection, worldviews

## Abstract

In spite of the social stigma surrounding them, conspiracy theories are a common topic of public debate on the Internet. The content and tone of these discussions provide a useful insight into the structure of conspiracist belief systems and the psychological characteristics of those who believe and disbelieve in conspiracy theories. In this focused review, we relate patterns of behavior found in online comments to the broader research literature on the psychology of conspiracy theories. Most notably, as conspiracism has its basis in disbelieving a mainstream or received narrative rather than in believing a specific alternative, most conspiracist arguments tend to fall along those same lines. Finally, we examine the implications of this methodology for future research into online discussion, particularly among hard-to-research populations.

## Introduction

By all accounts, **conspiracy theories** have been around for a long time. Contemporary speculation about assassinations, terrorist attacks, wars, medicine, and climate science follows on from a rich tradition—the witch panics of medieval Europe, rumors of Masonic conspiracies to destroy Christianity and monarchy, and long-standing suspicions that governments lie and cheat in order to maintain power. In conspiracist worldviews, the major forces in the world are not overt, but covert, hiding from the world at large as they move the pieces into place for their impending masterstroke (Hofstadter, 1964; Byford, 2011).

KEY CONCEPT 1 **Conspiracy theory**

An allegation regarding the existence of a secret plot between powerful people or organizations to achieve some goal (usually sinister) through systematic deception of the public.

The Internet has been a particular boon for the spread of conspiracy theories. Online publishing is free, instantaneous, global, and unburdened by editorial control. Conspiracy theories, now unhindered by skeptical publishers and producers, can reach a wider audience than ever—and one does not need to seek them out to come across them (Klein et al., 2015). They can be seen in the comment sections of many major news websites, for example, providing an underground counterpoint to the views expressed in the parent article (cf. Sapountzis and Condor, 2013; Harambam and Aupers, 2015). As the visibility of conspiracy theories has increased, so too has the volume of research into the psychology of conspiracy belief. Although there was some early work on the subject in the mid- to late-twentieth century, most of what we know about belief and disbelief in conspiracy theories comes from research conducted in the past 15 years. Much of this research is correlational, so it remains largely unclear whether the identified variables contribute to conspiracy belief, are effects of conspiracy belief, or are associated with it for some other reason. Nevertheless, a general picture is emerging, and given the right information we can predict with reasonable certainty the degree to which someone sees conspiracy as the driving force in human history.

In this focused review, we use the content of online discussions of conspiracy theories to place recent research findings into context. We review the methods and findings of a recent study (Wood and Douglas, 2013) that examined Internet debates regarding 9/11 conspiracy theories, drawing conclusions from the way in which people choose to represent their beliefs to others and relating the findings of this study to the broader research context.

Given what we know about conspiracist worldviews, what can we expect about how people might try to convince others of conspiracy theories? A core concept here is projection—the tendency to use the self as a model for how others think and behave. Many social judgments rely on projection, including belief and disbelief in conspiracy theories. The degree to which someone believes that a conspiracy is behind a particular event (say, the death of Princess Diana) depends in part on whether they would carry out the conspiracy themselves if they were in the same position as the alleged perpetrators (Douglas and Sutton, 2011). In other words, people may think “If I were in their position, I would have done it too,” so then it is plausible that such a conspiracy actually happened.

Projection is also used in persuasion. We cannot see into others' minds, and we do not always know someone well enough to predict how they will react to something. In the absence of information about others, we tend to assume that they are more or less like us, and that their reactions to a certain stimulus will be similar to our own—in other words, we project (for a review, see Robbins and Krueger, 2005). When we know relatively little about our audience, we use the arguments that we ourselves find most convincing, and these tend to be the arguments that fit with our own worldview (Darwin et al., 2011; Newheiser et al., 2011; Wood et al., 2012; Lewandowsky et al., 2013). This reasoning is the core of the current focused review. In the absence of information about what others might find convincing—say, when arguing with an audience of complete strangers over the Internet—our arguments will tend to reflect our own beliefs and preconceptions. Thus, an attempt at persuasion can serve as an informative window into the mental life of the persuader.

In a recently published study (Wood and Douglas, 2013), we made several predictions regarding the patterns of discourse in online arguments about conspiracy theories. Our hypotheses were based on the logic that in the absence of information about the target of persuasion, arguments are essentially projective. In other words, people's chosen arguments reflect their own belief systems.

Conspiracy theories regarding 9/11 presented themselves an ideal subject of study for this type of analysis. These theories generally allege that the 9/11 attacks were perpetrated by elements within the U.S. government, intelligence agencies, and/or Israel, and falsely blamed on Middle Eastern extremists. Common claims include that the Twin Towers and World Trade Center 7 were destroyed by demolition charges planted ahead of time, that the Pentagon was hit by a missile or drone rather than a passenger jet, and that United Airlines Flight 93 was shot down by a fighter jet or surface-to-air missile. While they are not a majority view in Western nations, unlike the alternative theories regarding the death of President John F. Kennedy (Swift, 2013), 9/11 conspiracy theories are reasonably common and highly visible in the public sphere. The 9/11 Truth Movement produces a tremendous amount of output, including books, documentary films, the peer-reviewed *Journal of 9/11 Studies*, conferences, websites, advertising campaigns, and so on. Its adherents include prominent religious figures and heads of state, and there are many different and competing versions of events that circulate within the movement. In short, the conspiracist community surrounding 9/11 is large, diverse, and well-developed. It is an excellent example of twenty first-century conspiracy culture.

While there are many discussion forums, blogs, and news websites devoted to both advancing and debunking the arguments of the 9/11 Truth Movement, we decided to avoid these venues as a source for data in our recent study (Wood and Douglas, 2013). There are a few reasons for this. First, we were primarily interested in persuasive communications—attempts at convincing another person of a particular interpretation of 9/11. In an environment where everyone more or less agrees that 9/11 was (or was not) an inside job, there is not likely to be much direct persuasive communication of this kind. Moreover, discussion in environments with a great deal of agreement among the participants can lead to group polarization—the tendency for like-minded groups and their members to develop more and more extreme opinions over time. The development of group polarization could in turn affect the level of hostility in communication (e.g., Spears et al., 1990). Finally, we considered it important to collect a reasonably large sample of both conspiracist and **conventionalist** comments, and websites catering to one group are unlikely to feature many postings from the other.

KEY CONCEPT 2 **Conventionalist explanation**

An account that attributes the cause of an event or social condition to overt processes or coincidence rather than to a hidden conspiracy.

As such, we extracted online comments from mainstream news websites. Although these sites inevitably take conventionalist positions, they attract large numbers of comments of both types, many of which are aimed at persuading undecided readers. Specifically, from four mainstream news websites, we collected all persuasive comments regarding 9/11 conspiracy theories from news articles on 9/11 from the second half of the year 2011. With the tenth anniversary of 9/11 happening that year, there were many articles on the attacks, their causes, their effects, and, of course, the conspiracy theories surrounding them. Whether the articles mentioned conspiracy theories or not, however, the comment sections would often include a spirited debate between conspiracists and conventionalists regarding the true cause of the attacks, providing a great deal of raw material.

In examining online debates about 9/11, we collected a large sample of 2174 comments from a variety of news websites. Our analysis of these comments revealed some noteworthy patterns regarding the nature of online discussion of conspiracy theories, and provided valuable insight into the minds of conspiracists and conventionalists. We measured several aspects of each comment, dealt with in each of the subsections below.

## Other conspiracy theories

In debating the various conspiracy theories regarding 9/11, it is relatively common for people to refer to other conspiracy theories, such as the assassination of President John F. Kennedy (JFK), as a reference point. Interestingly, the number of unrelated conspiracy theories mentioned favorably and unfavorably was different between conspiracist and conventionalist comments. On average, conspiracist comments mentioned about six times as many other conspiracy theories as being true as conventionalist comments did (0.12 vs. 0.02 per comment). Conventionalist comments, on the other hand, made about nine times as many negative references to other conspiracy theories as conspiracist comments did (0.18 vs. 0.02 per comment).

This replicates a classic finding in conspiracy psychology—the tendency for conspiracy beliefs to be positively correlated with one another. In the most comprehensive analysis of the correlates of conspiracy theorizing to date, Swami et al. (2010) were able to explain over 50% of the variance in beliefs in 9/11 conspiracy theories with a model incorporating a number of psychological variables. People were more likely to believe that 9/11 was an inside job if they were highly exposed to the relevant conspiracy theories, cynical about politics, disagreeable, and anti-authoritarian. The strongest predictor by far, however, was beliefs in other conspiracy theories. In fact, this is probably the most consistent finding of the research literature so far—the more someone believes in one conspiracy theory, the more they tend to believe in others (see also Goertzel, 1994; Swami et al., 2011; Wood et al., 2012). While this correlation may be attributable in part to the fact that many diverse conspiracy beliefs are predicted by the same variables (Sutton and Douglas, 2014), many researchers have interpreted this key finding as evidence for a general conspiracy worldview, a belief system in which conspiracy is the dominant force in history and the truth of major events is hidden from the public as a matter of course (Goertzel, 1994; Imhoff and Bruder, 2014). This worldview or thinking style, sometimes referred to as **conspiracist ideation** (e.g., Lewandowsky et al., 2013), is characterized by a general opposition to the mainstream or a distrust of the institutions of society at large. Conspiracist ideation is seen not as a positive belief, but a negative belief—a disbelief, or a generalized rejection of received narratives rather than an acceptance of specific alternatives. Being a “conspiracy theorist” is not about believing in a particular conspiracy, but in rejecting the official story.

KEY CONCEPT 3 **Conspiracism/conspiracist ideation**

The general tendency to attribute significant events or social conditions to hidden conspiracies rather than to overt processes or coincidence.

Several converging lines of evidence support this conception of the nature of conspiracist ideation. Wood et al. (2012) demonstrated that mutually contradictory conspiracy beliefs tend to be positively correlated—the more someone believed that Princess Diana was assassinated by MI6, the more they also tended to believe that she was assassinated by her boyfriend's family's business rivals; the more someone believed that Osama bin Laden died long before his supposed death in 2011, the more they believed that he survived the American raid that was said to have killed him. Imhoff and Bruder (2014) and Oliver and Wood (2014) have demonstrated that conspiracist ideation is mostly independent of other sociopolitical dimensions like right-wing authoritarianism, conservatism, and social dominance orientation (though not entirely; see, e.g., Grzesiak-Feldman and Izrycka, 2009). Van Prooijen et al. (2015) and Inglehart (1987) have shown that conspiracy belief tends to be higher among people who find themselves outside the political mainstream—those with either extreme left-wing or extreme right-wing politics are more likely than relative centrists to perceive a conspiracy behind society. Acceptance of conspiracy theories is also positively correlated with proneness to boredom (Brotherton and Eser, 2015), agency detection (Van der Tempel and Alcock, 2015; Douglas et al., in press), political cynicism (Swami et al., 2010, 2011), and **anomie** (Goertzel, 1994; Abalakina-Paap et al., 1999). Similarly, Swami et al. (2010) found that conspiracy belief is more prevalent among people with a disagreeable personality, though other studies have found no such effect (Swami et al., 2013; Lobato et al., 2014). By portraying a disliked outgroup as a sinister enemy of ambiguously vast power, conspiracy theories may help to manage threat and anxiety (Kofta and Sędek, 2005; Swami, 2012; Grzesiak-Feldman, 2013; Sullivan et al., 2014; Mashuri and Zaduqisti, 2015). Finally, theoretical and qualitative works present a convincing case that conspiracy theorizing is very often an anti-authoritarian activity, focused on challenging dominant societal power structures and providing counter-narratives to mainstream understandings of the world (Raab et al., 2013; Sapountzis and Condor, 2013; Harambam and Aupers, 2015).

KEY CONCEPT 4 **Anomie**

A feeling of alienation and disconnection from the ideology and values of society at large.

Taken together, this body of research suggests that the conspiracist mindset is at odds with acceptance of the mainstream. People who believe many conspiracy theories tend to feel alienated from society, to harbor extreme political views, to feel anxious or threatened, to be cynical about politics, to have generally disagreeable personalities, to hold views that diverge from the accepted mainstream, and to generally mistrust others. On this basis, it seems quite likely that the conspiracist mindset is characterized to a large extent by disbelief in official narratives rather than positive belief in alternatives, leading to a widespread acceptance of many different—and overtly unrelated—conspiracy theories.

The finding that conspiracist comments tend to be more positive than conventionalist comments about unrelated conspiracy theories matches this body of literature, and thereby strengthens our case that quantitative content analysis of online communication can provide valuable information regarding the thought processes of the communicator. When there is minimal information about the audience, as in this case, people use arguments that fit with their broader worldview and thinking style. The Internet is home to a tremendous amount of persuasive communications written in public. Such communications could prove to be an extremely rich source of observational data for future studies. Moving beyond comments on others' work, individual websites or videos could be coded according to a similar scheme. People seem intuitively less likely to make inferences about others' worldviews—thereby heightening their likelihood to project—when they are attempting to reach a broad and nonspecific audience, rather than responding to a particular person in a comment or forum post.

Interestingly, there was some variance in the other conspiracy theories chosen by commenters. For the most part, conspiracist comments referred to relatively well-known and comparatively mundane theories regarding assassinations and terrorist plots. The death of JFK was a popular topic, for instance—many conspiracist comments drew parallels between the widely-believed alternative theories of President Kennedy's assassination (Swift, 2013) and theories that 9/11 was likewise carried out in secret by the American power elite. Conventionalist comments, on the other hand, were much more likely to refer to exotic or ridiculous-sounding conspiracy theories, such as a cover-up of the existence of Bigfoot, alien abductions, or reptilian shapeshifters. This raises the possibility that the differences between conventionalist and conspiracist comments were an artifact of rhetorical congruency—naturally, people will pick examples that will support their arguments. However, the opposite approach is also a valid rhetorical strategy. A 9/11 conventionalist who believes that Princess Diana was assassinated may be a more convincing figure to a conspiracist than one who does not believe any conspiracy theories (Maass and Clark, 1984).

## Positive and negative argumentation

Based on indications from prior research that the conspiracist worldview has its basis in disbelief in official explanations rather than positive belief in alternatives (e.g., Wood et al., 2012), we expected to see a particular imbalance in the degree to which commenters used positive and **negative arguments**. Specifically, we predicted that conspiracist comments would contain fewer positive and more negative arguments than conventionalist comments. In agreement with our prediction, conventionalist comments used **positive arguments** about 56% of the time, compared to 31% of conspiracist comments. Negative arguments showed the opposite difference—only 44% of conventionalist comments argued against the opposing interpretation, while 64% of conspiracist comments did the same.

KEY CONCEPT 5 **Negative arguments**

Advancing a position by presenting an argument that contradicts an opposing position: “9/11 was clearly an inside job, since the official story can't explain the collapse of Building 7.”

KEY CONCEPT 6 **Positive arguments**

Advancing a position by presenting an argument that directly supports it: “9/11 was clearly an inside job, since thermite residue in the wreckage of the Twin Towers is conclusive evidence of a controlled demolition.”

Unlike conventionalists, who mostly provided arguments in favor of their own position, conspiracists overwhelmingly argued against the opposing positions. By our reasoning above, this choice of communicative strategy is not accidental. Rather, it is the sort of argument that conspiracists themselves find most persuasive and it therefore gives a clue as to how they think about the world. The use of negative over positive arguments supports the idea explored above, that the conspiracist worldview is not about belief in particular explanations for events but a *disbelief* in particular explanations—specifically, in mainstream, received, or official accounts of major events or social conditions. For the most part, alternative accounts of 9/11 and events like it are not initially accepted by conspiracists because they do a good job of explaining the available facts, but because they oppose the account that comes from a disliked or distrusted source and because a conspiracist account fits with a broader conspiracist worldview. Someone who prefers a conspiracist explanation for 9/11 may not have a specific alternative account in mind at all (cf. Dean, 2002). Rather, if the official account can be discounted, a conspiracy—however, vague—must have been at work. Recent research continues to bear out this general idea. Van Prooijen and Jostmann (2013) have demonstrated the importance of feelings of uncertainty in cultivating conspiracy belief; Einstein and Glick (2014) have shown that specific elaboration upon conspiracy theory claims appears to decrease belief in them; and Swami et al. (2014) have demonstrated that adopting an analytical mindset, one characterized by attention to detail, tends to attenuate conspiracy theory belief. Also, when conspiracy theories are elaborated upon, people with a better grasp of probability and logical reasoning may reject them; correlational research has shown lower conspiracy belief in people who are less susceptible to the conjunction fallacy (Brotherton and French, 2014), and lower belief amongst individuals who are less likely to attribute agency and intentionality to environmental factors by default (Douglas et al., in press).

Internal politics might also be a factor in the decision to use negative rather than positive arguments to promote conspiracy theories. As noted above, the 9/11 Truth Movement is extremely diverse. Other than the mere existence of a conspiracy and the falsity of the mainstream account, there may be little that everyone in the movement agrees upon. Conflicts within the Truth Movement over topics such as the motives, perpetrators, or details of the execution of the attacks can be intense, and often involve allegations that the other party is working for the conspirators, pushing a misleading conspiracy theory in order to throw well-meaning truth-seekers off the trail (e.g., Wood, 2009; Fox, 2013). Negative arguments avoid this pitfall. All 9/11 conspiracists can agree that the conventional account is false, so criticizing it is uncontroversial. A tendency toward a negative argument style allows conspiracists to focus on arguing more with conventionalists and less with each other.

These findings prompt some key questions about the effectiveness and prevalence of positive and negative argumentation. If someone prefers positive to negative arguments or vice versa, is it because they find their preferred type of argument most convincing, or because they find their preferred argument type more accessible and easier to generate? Does the prevalence of negative argumentation in the conspiracy theory world owe its existence to the structure of the conspiracist belief system or to political considerations and a desire for a “big tent” of conspiracy theory? Finally, are positive or negative arguments ultimately more successful? Does it depend on the subject matter? Answering these questions may provide instructive insights into developing effective persuasive communications tailored to their recipients' worldviews, and inform efforts by policy-makers to counter the spread of potentially harmful conspiracy theories, such as those concerning vaccination or global climate change (cf. Jolley and Douglas, 2014a,b; Douglas and Sutton, 2015; Douglas et al., 2015, in press).

## Mistrust and powerlessness

Only two comments in the sample of 2174 contained expressions of powerlessness, an insufficient amount for any reasonable analysis. Past research has shown strong connections between conspiracy belief and feelings of powerlessness or loss of control (Hamsher et al., 1968; Abalakina-Paap et al., 1999; Whitson and Galinsky, 2008). However, powerlessness and a lack of control are not attractive traits to have when attempting to convince others to one's worldview. Expressions of powerlessness may have been in short supply in this sample not because feelings of powerlessness are rare, but because they were suppressed by the demands of the situation.

Expressions of mistrust, however, were not in short supply. Conspiracist comments were more likely to express mistrust (of society, groups, institutions, and specific people) than conspiracist comments were (10.6 vs. 1.4%). This result was as expected. A great deal of research into interpersonal trust indicates that low trust is associated with beliefs in conspiracy theories (Hamsher et al., 1968; Wright and Arbuthnot, 1974; Goertzel, 1994; Yelland and Stone, 1996; Abalakina-Paap et al., 1999; Leman and Cinnirella, 2013). Whether trust is a cause, an effect, or both, the relationship seems intuitive. People who trust others are more likely to accept received explanations at face value, and less likely to suspect ulterior motives; likewise, belief that large-scale conspiracies are the driving force in society would not foster a trusting worldview. Projection may also be a factor in interpersonal distrust, since people who rate themselves as more likely to conspire are suspicious that others might do the same (Douglas and Sutton, 2011).

## Hostility

We rated each comment for hostility on a scale from 1 to 5, and found that conventionalist comments tended to have a higher hostility rating than conspiracist comments (2.08 vs. 1.44 on a 1–5 scale).

Decades of work on the psychology of social influence has shown that minorities are most effective in convincing others of their views if they are calm, consistent, and informative (e.g., Latané, 1981). Majority groups, on the other hand, can enforce adherence to social norms in a more forceful manner. Opinion polls show that most people in the West do not believe that 9/11 was an inside job (WorldPublicOpinion.org, 2008)—the conspiracist view is a minority one. Conventionalists, being in the majority, have more flexibility in this regard. While conventionalists can attempt to enforce conformity to the majority viewpoint, conspiracists must provide novel information and attempt to produce internal attitude change rather than outward conformity (Latané, 1981). The lowered hostility found for conspiracists indicates that they may have internalized some of the properties of good minority influencers.

**Figure 1:**
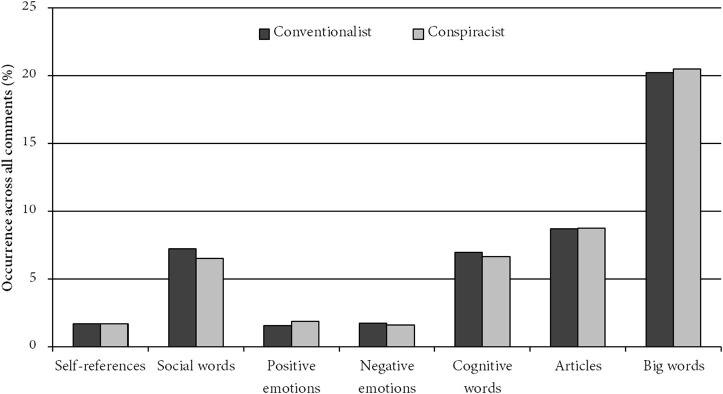
**Results from LIWC analysis of comment database, showing prevalence of self-references (I, me, my), social words, positive emotions, negative emotions, cognitive words, articles, and words with more than six letters**.

Due to the counterintuitive nature of the hostility finding, in a novel analysis for the present article we followed up on our coding with a run of the Linguistic Inquiry and Word Count (LIWC) textual analysis software (Pennebaker et al., 2001, 2007). Combining all conspiracist comments together revealed an instructive difference from a combined entry of all conventionalist comments: while the two were comparable in most cases (see Figure 1), the conspiracist comments used more positive than negative emotional words (1.85% positive vs. 1.58% negative), while the opposite was true for conventionalist comments (1.55% positive vs. 1.74% negative). While this analysis did not reveal the targets of these emotional expressions, this finding is all the more remarkable as the conspiracist comments usually posited a massive, murderous conspiracy—something that would seem to justify a great deal of negativity. The fact that conspiracists' negativity was outweighed by conventionalists' is noteworthy.

This finding is somewhat counterintuitive. Conspiracy explanations for events are often stigmatized as the product of mental illness or gullibility (Bratich, 2002, 2008). Of course, conspiracists can be quite hostile as well, often accusing their rhetorical opponents of naiveté or even complicity in the conspiracy (Crane, 2008; Byford, 2011), but in this case the former seems to outweigh the latter. Interestingly enough, however, this finding seems to contradict a previous finding that conspiracy belief is positively correlated with trait hostility (Abalakina-Paap et al., 1999). It also runs counter to the stereotype of conspiracists as behaving in an irrational and unbalanced manner (Bratich, 2008). However, anonymous online communication is a fairly specific situation, and it is difficult to draw firm conclusions from these results regarding personality traits or more broad tendencies in social interaction. It is quite possible that conspiracist views are correlated with higher trait hostility in general, but that something about the communicative situation provokes an unusually hostile reaction among conventionalists. Regardless, this result should sound a note of caution for conventionalists: hostility is best avoided when trying to convince others of something. Conventionalists tend to overestimate their own rationality and underestimate others', which, as Klein et al. (2015) note, may be responsible for conspiracy theories' unexpected and unnoticed influence upon those who initially reject them (Douglas and Sutton, 2008).

## Usage of “conspiracy theory”

Data analysis indicated a general tendency to avoid the conspiracy theory label. Whether conspiracist or conventionalist, commenters were largely unwilling to apply it to their own beliefs, and would often argue the point if others did so. Evidently, despite endorsing conspiracist explanations, and being sufficiently committed to them to argue about them extensively, people appeared to be motivated to avoid the social stigma associated with the label. In objecting to the label, many commenters characterized it as an intellectual slur used to marginalize dissent and pathologize reasonable suspicion, which is in line with recent scholarly characterizations by Bratich (2002, 2008), deHaven-Smith (2013), and Husting and Orr (2007). Recent research has borne out the intuitive idea that such a stigma exists, demonstrating that people tend to view conspiracists as gullible, naive, crazy, and dishonest (Klein et al., 2015). However, whether the label is an effective rhetorical weapon is unclear; recent experiments have shown that simply labeling something a conspiracy theory does not reduce belief in it (Wood, 2015). This is a prime area for future research.

## Discussion

In general, the findings of Wood and Douglas (2013) provide an instructive insight into the conspiracist mindset. The results are in agreement with past work on conspiracy theories, which indicates that the methodology is a sound one. The arguments that people use may be a reflection of what they find most convincing, which in turn may reveal something about their psychological state.

Content analysis of online communication has several potential advantages. The amount of raw material publicly available for such analysis on the Internet is huge. This method also allows investigation of populations that would be averse to filling out questionnaires or participating in laboratory experiments; in fact, people with a high degree of conspiracist ideation are probably such a category. There are, of course, some pitfalls to be avoided as well. Namely, there is some degree of self-presentation at work, as seen in the case of the powerlessness result reviewed above. In addition, the logic of the analysis depends on people using projection to determine which persuasive strategies to adopt. In situations where the person producing the communication knows their audience, they will tailor their message accordingly. In that case, the content of the message would be a reflection of the writer's perception of the audience as well as the writer's own psychological state—it is only when there is little or no information about the audience that communications are based mostly on projection (Friestad and Wright, 1999; Douglas et al., 2010; Vogel et al., 2010). Researchers must therefore take care to establish whether the communications under analysis are written to a general (and thus projected) audience, or to a specific person known to the author. Moreover, social desirability can play a role in persuasive communication. Feelings of powerlessness are not unheard-of among either conspiracists or conventionalists, yet almost no commenters were willing to state that they felt powerless. This may be because feeling powerless is not an attractive prospect when trying to sell others on adopting a worldview. Given the self-presenting nature of persuasive communication, caution is warranted when drawing conclusions regarding socially undesirable subject matter. It is also worth noting that we have interpreted our results, especially the hostility result, as manifestations of the influences exerted by the situation and the properties of the commenters' belief systems. However, there is a robust literature on individual differences in conspiracy belief (e.g., Swami et al., 2011; Uscinski, 2014), so it is possible, as in any correlational design, that the arrow of causation points the other way.

Finally, in any analysis of online discussion, researchers must take care not to over-interpret the findings. We extracted about twice as many conspiracist as conventionalist comments, but due to the self-selecting nature of online discussion this does not match the popularity of 9/11 conspiracy theories in the broader population (WorldPublicOpinion.org, 2008). Moreover, there is the potential for prolific individuals to dominate conversation. Although the sample consisted of 2174 comments, these were written by only 1156 unique authors, 321 of whom commented more than once. While our findings were the same when the unit of analysis was authors rather than comments, this will not always be the case, and there is always the potential for “sock puppets,” or alternate online identities run by the same person, to interfere with such calculations by creating the appearance of consensus where there is none. In general, researchers should be aware of the peculiarities and pitfalls of Internet commenting culture in order to draw reliable conclusions from an online comment sample.

## Conclusions

In this project, we have reviewed the findings of a previous study (Wood and Douglas, 2013) in the context of existing research into the psychology of conspiracy theories. Consistent with what we expected from prior research, conspiracist comments (relative to conventionalist comments) were less hostile, more likely to make negative arguments, less likely to make positive arguments, and more positive toward unrelated conspiracy theories. These findings support the idea of a broad conspiracist worldview based on disbelief in mainstream or received narratives, and highlight the analysis of online communication as a potentially useful tool for gaining insight into the worldview and assumptions of the persuader.

### Conflict of interest statement

The authors declare that the research was conducted in the absence of any commercial or financial relationships that could be construed as a potential conflict of interest.
